# Risk factors for ocular hypotony after XEN Gel Stent implantation

**DOI:** 10.1007/s00417-022-05831-2

**Published:** 2022-10-06

**Authors:** Maria E. Galimi, Julia M. Weller, Friedrich E. Kruse, Robert Laemmer

**Affiliations:** 1 Department of Ophthalmology, University Hospital Erlangen, Erlangen, Germany; 2 Department of Ophthalmology, Friedrich-Alexander University Erlangen-Nürnberg, Erlangen (FAU), Schwabachanlage 6, 91054 Erlangen, Germany

**Keywords:** Glaucoma, Minimal invasive glaucoma surgery (MIGS), XEN Gel Stent, Hypotony

## Abstract

**Purpose:**

To investigate the incidence of postoperative hypotony, and risk factors for the development of hypotony in eyes who had undergone XEN Gel Stent implantation.

**Methods:**

In this retrospective, single-centre case series, medical records of 170 consecutive eyes who had undergone XEN Gel Stent implantation with or without simultaneous phacoemulsification for primary or secondary open angle glaucoma were analysed. Primary outcome parameters were the incidence of postoperative hypotony and potential risk factors for its development, and secondary parameters were pre- and postoperative visual acuity, intraocular pressure (IOP), and number of IOP-lowering eye drops.

**Results:**

Postoperative hypotony ≤ 6 mmHg occurred in 57% of eyes. Hypotony was without complications in 70.1%, 13.4% had transient complications with spontaneous resolution, and 16.5% had complications requiring treatment. Mean visual acuity logMAR before surgery accounted for 0.47 ± 0.46 in all eyes and 0.47 ± 0.48 at the 4-week visit. There was no significant difference of BCVA in the group of eyes with and without postoperative hypotony before and after surgery. The mean IOP before surgery was 24.6 ± 8.4 mmHg and decreased significantly to 18.4 ± 10.2 after 4 weeks.

Eyes with an axial length over 24.3 mm had a threefold increased risk for postoperative hypotony (OR 3.226, 95% confidence interval 1.121–9.279). This risk was decreased in eyes with simultaneous cataract surgery (OR 0.483, 95% confidence interval 0.258–0.903).

**Conclusion:**

In our sample, postoperative hypotony was a common complication after XEN Gel Stent implantation, but serious, persistent complications were rare. A longer axial length predisposes the eye for the development of hypotony.
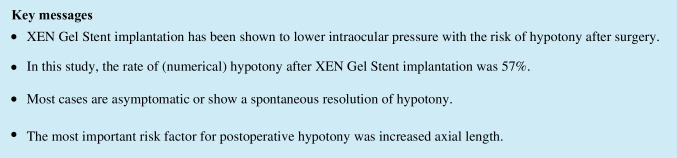

## Introduction

Glaucoma is one of the most common vision-threatening ocular diseases. Lowering of the intraocular pressure is the main therapeutical strategy. The gold standard of surgeries to reach this goal has been trabeculectomy for several decades [1].

However, possible postoperative complications, such as bleb-leaks, hypotony due to overfiltration, scarring of the bleb, and consequently underfiltration, up to endophthalmitis are well known, as well as the long, intensive postoperative care that is needed [1–4].

Minimal invasive glaucoma surgery (MIGS) has been developed in the last years to reduce complications. It is performed by a less invasive “ab interno” approach, which preserves the conjunctiva [1, 5, 6].

One important example of MIGS is the XEN Gel Stent implant, a 6 mm long, 45 µm wide (internal lumen diameter) hydrophilic tube of porcine gelatine, which can “ab interno” be placed in the anterior chamber and allows the aqueous humour to be filtered in the subconjunctival space [7, 8].

Without the need of a valve, this implant should avoid overfiltration and in this way early postoperative hypotony [8].

Nevertheless, early postoperative hypotony and its consequences (choroidal haemorrhage, choroidal detachment, flattening of the AC) are still an issue after XEN Gel Stent implantation.

The purpose of our study is to define the incidence of early postoperative hypotony and to investigate possible patient-specific risk factors.

## Materials and methods

### Patients

In this retrospective single-centre study, eyes that received a XEN Gel Stent implantation with or without combined cataract surgery were analysed. The surgeries have been performed by a single surgeon (RL) at the Department of Ophthalmology at the Friedrich-Alexander University of Erlangen Nuremberg.

The medical files of all 170 eyes (136 patients) which have undergone XEN Gel Stent implantation in the time between September 2015 and March 2020 were analysed. Thirty-four patients had received XEN Gel Stent surgery in both eyes.

Eyes with a previous XEN Gel Stent implantation at our clinic were included in this study. There were no exclusion criteria in order to present real-life data without bias by removal of difficult eyes. Patients had undergone surgery if the target IOP level was not reached either due to exhausted medical treatment or due to intolerance of eye drops. Target IOP was defined individually as suggested by the European Glaucoma Society on the basis of progression of disease.

The Institutional Review Board (IRB)/Ethics Committee of the Friedrich-Alexander University Erlangen Nurnberg approved the study (Approval ID: 79_21 Bc). The study was in adherence to the tenets of the Declaration of Helsinki. Informed consent to surgery was obtained from all patients prior to surgery.

### Surgical technique

After adequate skin disinfection and preoperative anaesthesia, the conjunctiva is marked superonasally in 3 mm distance to the limbus. This is where the end of the stent is expected to exit after the implantation. Mitomycin C 0.01 mg is then injected subconjunctivally and massaged in the area of implantation. Two side incisions are made superotemporally and inferotemporally. The anterior chamber is filled with a cohesive viscoelastic (Z-Healon™plus 0.55 ml, Zeiss) for stabilization. Afterwards, the surgeon inserts the XEN Gel Stent through the inferior incision and advances it carefully to the superonasal chamber angle. The XEN Gel Stent is delivered through the trabecular meshwork and into the subconjunctival space. After microscopical check of the correct stent position, the viscoelastic is removed from the anterior chamber using an aspiration/irrigation system, followed by hydration of the incisions. A prominent bleb superonasally should be seen.

In the surgeries performed from October 2017 on, Dispasan (Z-Healin® Cohesive OVD, 10 mg/ml, Zeiss) is instilled into the anterior chamber at the end of surgery to stabilize it and to avoid early postoperative hypotony.

The postoperative topical treatment consisted of preservative-free ofloxacin 3 mg/ml eye drops 3 times a day for 1 week, and preservative-free dexamethasone 1.0 mg/ml eye drops every hour. Dexamethasone eye drops were tapered slowly over at least 3 months.

In case of hypotony with complications and without spontaneous resolution (*n* = 16), the AC was filled with cohesive viscoelastic. Indication for this intervention was made in hypotonic eyes with vision-threatening, persistent hypotonic complications (shallow AC, choroidal detachment with imminent kissing choroids). Other treatment modalities were not necessary (e.g. stent removal and vitrectomy).

### Main outcome parameters

The main outcome parameter of the study was the incidence of postoperative hypotony. Numerical hypotony was defined as IOP ≤ 6 mmHg. Secondary parameters were pre- and postoperative IOP values, number of anti-glaucomatous eye drops before and after surgery, best-corrected visual acuity (BCVA), and incidence of complications. Choroidal swelling, hypotonic maculopathy, AC haemorrhage, and leakage from the bleb (stent extrusion) were defined as postoperative complications. Choroidal swelling, hypotonic maculopathy, and a shallow AC were defined as symptomatic hypotony, whereas AC bleeding and stent extrusion were not considered as hypotonic-related complications. The outcome parameters were correlated with possible risk factors (axial length, myopia, arterial hypertension).

Intraocular pressure was measured using Goldmann applanation tonometry. The measurement of the axial length was performed with the IOL master 500 (Zeiss®).

### Statistics

The program SPSS for Windows (version 24, SPSS Inc., Chicago, IL, USA) was used for statistical analysis of the data.

Normal distribution of data was tested with the Kolmogorov–Smirnov test showing no normal distribution of the data. The nonparametric Mann–Whitney *U*-test was used for comparison of results between groups. A two-sided *p* value of < 0.05 was considered as statistical significant. Categorical data were compared with the chi-square test. For the comparison of preoperative and postoperative parameters (repeated measurement of BCVA and IOP), the nonparametric Wilcoxon sign rank test was used.

## Results

### Patients’ characteristics before surgery

The patients’ characteristics are listed in Tables 1 and 2 regarding age at time of surgery, sex, diagnosis, systemic risk factors (arterial hypertension), and anatomical features (axial length, pachymetry, and refraction).

**Table 1:** Patient’s characteristics

	All eyes	Hypotony < 6	No hypotony	*p* value (chi-quadrat, 2-seitig)
Diagnosis* (*n*) [%]:				
-pOAG -sOAG • PEX • Melanindispersion • Inflammatory • Other	74 [44%] 96 [56%] 65 [38%] 8 [5%] 9 [5%] 14 [8%]	40 [41%] 57 [59%] 38 [39%] 6 [6%] 4 [4%] 9 [9%]	34 [47%] 39 [53%] 27 [37%] 2 [3%] 5 [7%] 5 [7%]	0.534
Arterial hypertension** (*n*) [%]:	77 [57%]	48 [59%]	29 [53%]	0.484
Sex** (*n* = male: female) [%]	71 [52%]: 65 [48%]	45 [55%]: 36 [45%]	26 [47%]: 29 [53%]	0.384

(*pOAG*, primary open angle glaucoma; *sOAG*, secondary open angle glaucoma; *PEX*, pseudoexfoliation)

^*^Numbers/percentages refer to the number of *eyes* (*n* = 170) included

^**^Numbers/percentages refer to the number of *patients* (*n* = 136) included

**Table 2:** Patient’s characteristics

	All eyes	Hypotony < 6	No hypotony	*p* value (Mann–Whitney *U*-test)
Age (mean ± SD)	67 ± 14	66 ± 15	68 ± 12	0.546
Spherical equivalent (diopters, mean ± SD)	− 0.99 ± 2.70	− 1.06 ± 3.05	− 0.87 ± 1.97	0.449
Pachymetry (µm, mean ± SD)	542 ± 50	539 ± 48	546 ± 53	0.429
Axial length (mm, mean ± SD)	23.65 ± 1.24	23.99 ± 1.29	23.30 ± 1.10	0.010

(*SD*, standard deviation)

The mean age at time of surgery was 67 ± 14 years (range 21–94 years). The male/female ratio was 52%/48% (*n* = 71/65). 77/136 patients (57%) had a positive medical history of arterial hypertension.

The excavation of the optic disc was graded using the Jonas classification [9]: stage 1 was found in 34 eyes (20%), stage 2 in 28 eyes (16.5%), stage 3 in 47 eyes (27.6%), and stage 4 in 61 eyes (35.9%) at time of surgery.

The stage of glaucoma was classified based on stated automatic perimetry (Octopus Perimetry–Haag Streit). At baseline, preperimetric glaucoma (MD ≤ 2 dB) was found in 5.9%, early stage glaucoma (MD > 2 dB ≤ 6 dB) in 15.4%, moderate glaucoma (MD > 6 dB ≤ 12 dB) in 29%, advanced glaucoma (MD > 12 dB ≤ 20 dB) in 27%, and end-stage glaucoma (MD > 20 dB) in 22.7%.

In 85.9% of eyes (*n* = 146), IOP-lowering eye drops were used preoperatively. Due to intolerance of anti-glaucomatous eye drops, 14.1% (*n* = 24) of eyes had no topical treatment at time of surgery. 5.5% of eyes under topical anti-glaucomatous therapy (*n* = 8) had one eye drop, 21.2% of eyes under topical anti-glaucomatous therapy two eye drops (*n* = 31), 43.2% of eyes under topical anti-glaucomatous therapy (*n* = 63) three eye drops, and 30.1% of eyes under topical anti-glaucomatous therapy (*n* = 44) four different eye drops.

Among them, betablocker eye drops were used in 114 eyes (78.1% of eyes under topical anti-glaucomatous therapy), prostaglandin-analogues in 124 eyes (84.9% of eyes under topical anti-glaucomatous therapy), alpha-agonists in 82 eyes (56.2% of eyes under topical anti-glaucomatous therapy), and carbonic anhydrase inhibitors in 117 eyes (80.1% of eyes under topical anti-glaucomatous therapy). 36.5% of all eyes (*n* = 62 of 170 eyes) were treated with acetazolamide systemically before surgery, either additionally to IOP-lowering eye drops in case of IOP decompensation or as only treatment in eyes with intolerance of anti-glaucomatous eye drops.

A total of 124 eyes (72.9%) had a history of at least one ocular surgery before the XEN Gel Stent implantation.

Fifty-seven eyes (33.5%) had received a laser intervention previously: 18 eyes (10.6%) had received a YAG-Iridotomy, and 39 eyes (22.9%) a selective laser trabeculectomy (SLT).

Twenty eyes (11.8%) had a history of glaucoma surgery: of these eyes, 5 (2.9% of all eyes) had a previous i-Stent inject implantation, 13 (7.6% of all eyes) a previous XEN Gel Stent implantation, and 2 (1.2% of all eyes) a Cypass implantation.

Other surgeries before XEN Gel Stent implantation comprised cataract surgery (phacoemulsification) in 66 eyes (38.8% of all eyes) and pars plana vitrectomy in 3 eyes (1.8% of all eyes). The reasons for vitrectomy were epiretinal membrane (*n* = 2) and the luxation of an intraocular lens (*n* = 1).

No eye was aphakic at the time of XEN Gel Stent implantation.

Thirty-four patients had bilateral XEN Gel Stent implantation. The rate of hypotony in the second eye of patients who had hypotony in the first eye (*n* = 17) was 53% (*n* = 9 eyes).

### Best-corrected visual acuity

Mean BCVA (logMAR) before surgery accounted for 0.47 ± 0.46 in all eyes, and decreased to 0.62 ± 0.44 after 1 week (*p* < 0.001, Wilcoxon test). At the visit 4 weeks after surgery, mean BCVA was 0.47 ± 0.48, which is similar to the preoperative value (*p* = 0.612, Wilcoxon test). There was no significant difference of BCVA in the group of eyes with and without postoperative hypotony (Table 3).

**Table 3:** Best-corrected visual acuity

	All eyes	Hypotony < 6	No hypotony	*p* value (Mann–Whitney *U*-test)
BCVA preoperatively (logMAR, mean ± SD)	0.47 ± 0.46	0.47 ± 0.42	0.47 ± 0.51	0.774
BCVA after 1 week (logMAR, mean ± SD)	0.62 ± 0.44	0.64 ± 0.37	0.60 ± 0.52	0.161
BCVA after 4 weeks (logMAR, mean ± SD)	0.47 ± 0.48	0.51 ± 0.49	0.42 ± 0.45	0.092

(*BCVA*, best-corrected visual acuity; *SD*, standard deviation)

### Intraocular pressure and use of IOP-lowering eye drops

**Fig. 1:**
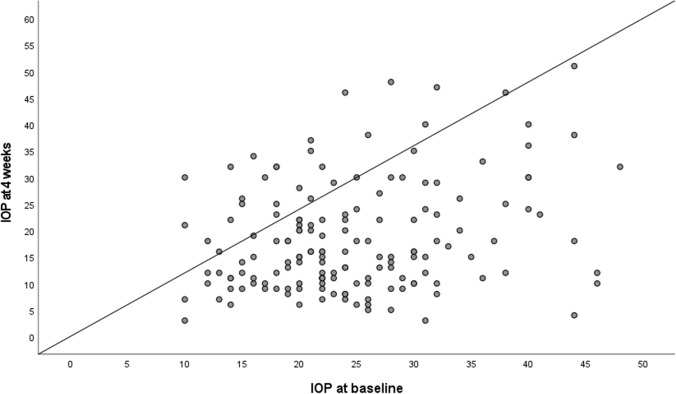
Scatter plot showing IOP data at baseline and at the 4-week visit

The mean intraocular pressure (IOP) before surgery was 24.6 ± 8.4 mmHg (range 12.0 to 48.0 mmHg) (Table 4). At the follow-up 4 weeks after surgery, mean IOP was significantly reduced to 18.4 ± 10.2 (*p* < 0.001) (Fig. 1).

**Table 4:** Intraocular pressure

	All eyes	Hypotony < 6	No hypotony	*p* value (Mann–Whitney *U*-test)
IOP preoperatively (mmHg, mean ± SD)	24.6 ± 8.4	23.5 ± 8.8	25.9 ± 7.7	0.039
Lowest IOP postoperatively (mmHg, mean ± SD)	6.0 ± 3.8	3.7 ± 1.7	9.1 ± 3.7	0.000
IOP after 4 weeks (mmHg, mean ± SD)	18.4 ± 10.2	17.7 ± 10.1	19.3 ± 10.4	0.381
IOP after 12 months (mmHg, mean ± SD)	15.4 ± 6.0	14.2 ± 4.8	17.09 ± 7.1	0.012

(*IOP*, intraocular pressure; *SD*, standard deviation)

At the 4-week visit, there was no significant difference of IOP between both groups (*p* = 0.381).

The mean number of IOP-lowering eye drops was 2.6 ± 1.3 before surgery in all eyes, and decreased to 0.1 ± 0.6 after 1 week, and 0.5 ± 1.2 four weeks after surgery (Table 5).

**Table 5:** Number of eye drops

	All eyes	Hypotony < 6	No hypotony	*p* value (Mann–Whitney *U*-test)
Number of eye drops preoperative (mean ± SD)	2.6 ± 1.3	2.6 ± 1.3	2.6 ± 1.3	0.698
Number of eye drops at 1 week (mean ± SD)	0.1 ± 0.6	0.1 ± 0.5	0.2 ± 0.6	0.267
Number of eye drops after 4 weeks (mean ± SD)	0.5 ± 1.2	0.6 ± 1.2	0.5 ± 1.1	0.887
Number of eye drops after 12 months (mean ± SD)	0.8 ± 1.2	0.9 ± 1.3	0.8 ± 1.2	0.819

(*SD*, standard deviation)

Numerical ocular hypotony, defined as IOP ≤ 6 mmHg, occurred in 57.0% of eyes (*n* = 97). There was no significant difference of the patient-specific parameters regarding the rate of postoperative hypotony except for axial length.

The number of IOD-lowering eye drops decreased in both other groups as well (Table 5): in the no hypotony group, the mean number preoperative was 2.6 ± 1.3, which decreased to 0.5 ± 1.1 after 4 weeks. In the hypotony group, the mean number of IOP-lowering eye drops was 2.6 ± 1.3 preoperative, and 0.6 ± 1.2 four weeks after surgery.

The mean interval between surgery and occurrence of hypotony was 1.3 ± 0.8 days (range 0–7 days) after surgery.

In Table 6 is shown the relationship between the development of hypotony and the status of the lens at the time of surgery (phakic eyes with XEN Gel Stent implantation only versus standalone operations in already pseudophakic eyes versus phakic eyes that underwent a combined surgery).

**Table 6:** Correlation between hypotony and status of the lens

	Standalone XEN implantation on phakic eyes (*n* = 37)	Standalone XEN implantation on already pseudophakic eyes (*n* = 66)	XEN implantation + cataract surgery (*n* = 67)	*p* value
Frequency of hypotony	26 (70.3%)	40 (60.6%)	31 (46.3%)	0.046
Asymptomatic hypotony	20	27	21	
Transient symptomatic hypotony	4	5	4	
Symptomatic hypotony requiring treatment	2	8	6	

There was no statistical significance in the development of shallow AC or choroidal swelling and the simultaneous XEN with phacoemulsification compared with XEN implantation only (*p* = 0.813).

### Long-term data

**Fig. 2:**
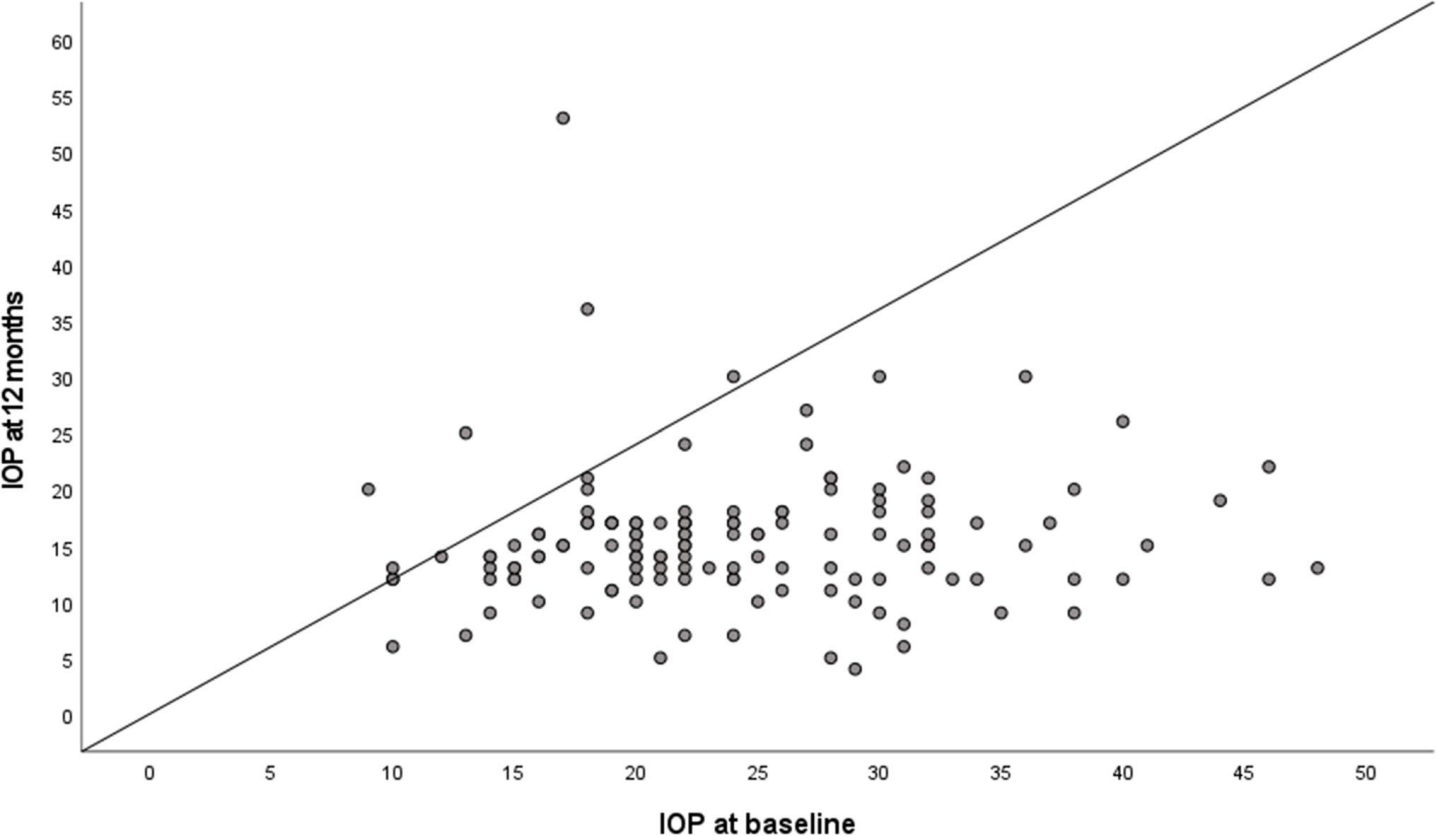
Scatter plot showing IOP data at baseline and at the 12-month visit

At the 1-year follow-up, mean IOP was 15.4 ± 6.0 mmHg (*n* = 135 eyes of 108 patients; *p* < 0.001 compared to baseline, Fig. 2). The mean number of anti-glaucomatous eye drops was 0.8 ± 1.2 at 12 months. There was no statistic significant progression of visual field loss after 1 year (MD at baseline 12.9 ± 7.4 dB; MD after 1 year 12.3 ± 7.2 dB; *p* = 0.728 Wilcoxon test).

A total of 11.9% (*n* = 16) of eyes had an IOP above 20 mmHg after 12 months making another glaucoma surgery or additional IOP-lowering eye drops necessary. Ten eyes did not reach the individual set target IOP and needed further glaucoma surgery.

### Complications

Postoperative complications comprised choroidal swelling (*n* = 25) and shallow AC (*n* = 4). Other complications described in the literature after XEN Gel Stent implantation did not occur in our study cohort (infection, hypotonic maculopathy). Complications occurred only in the group with hypotony compared with normotonic eyes.

Among the hypotonic eyes (*n* = 97), the hypotony was without complications (as defined above) in 68 eyes (70.1% of hypotonic eyes, 40% of all eyes). Thirteen eyes (13.4% of hypotonic eyes, 7.6% of all eyes) had transient complications with spontaneous resolution, and 16 eyes (16.5% of hypotonic eyes, 9.4% of all eyes) had complications requiring treatment. Twenty-five eyes had a choroidal swelling, and 16 of them had to receive a treatment. Interestingly, all eyes that had a shallow AC (*n* = 4) had a choroidal swelling as well, and needed treatment.

### Safety and adverse events

In ten eyes, the target IOP was not reached and needed further glaucoma surgery within the follow-up period of 1 year: six eyes underwent repeat XEN Gel Stent implantation, three eyes received a suprachoroidal stent (Cypass), and one eye underwent trabeculectomy. Another two eyes had stent extrusion requiring revision. Nine eyes had anterior chamber haemorrhage with spontaneous resolution. One eye had massive intraocular haemorrhage after XEN Gel Stent implantation requiring vitrectomy.

The rate of postoperative hypotony in the second eye was not increased compared to the hypotony rate of the general cohort: 34 patients had undergone a bilateral XEN Gel Stent implantation. Seventeen eyes were hypotonic after surgery of the first eye, whereof 9 eyes (53%) developed hypotony in the second eye, too.

### Subgroup analysis: impact of viscoelastic at the end of surgery

From October 2017 on, the surgical technique has been changed by the instillation of a cohesive viscoelastic in the AC in order to avoid early postoperative hypotony.

Among the 170 eyes analysed in this study, 42 (24.7%) received Z-Healin® in the AC. Hypotony occurred in 55.5% (*n* = 71) of the eyes without viscoelastic (41.8% of all eyes), and in 62% (*n* = 26) of eyes with viscoelastic at the end of surgery (*p* 0.480, Fisher’s exact test).

In the eyes filled with viscoelastic, the mean interval between surgery and onset of hypotony was 1.4 ± 1.4 days, in the eyes without viscoelastic, 1.2 ± 0.5 days, respectively (*p* = 0.576). The lowest IOP value among the eyes with viscoelastic was 5.4 ± 3.5 mmHg, and in the group without viscoelastic was 6.2 ± 4.0 mmHg (*p* = 0.238).

### Influence of axial length on incidence of hypotony

Mean axial length (mm) was 23.65 ± 1.24 in the entire study cohort (range: 21.21–27.27 mm). In the group of eyes with postoperative hypotony, AL was 23.99 ± 1.29 mm, in the group without hypotony23.30 ± 1.10 mm, respectively (*p* = 0.010) (Table 2).

For the analysis of risk factors for the occurrence of these complications, a subgroup analysis was performed: since axial length had been found to be significantly different in both groups (with vs without hypotony), we divided the range of axial length values into four quartiles (Table 7). The eyes with the longest axial length (4th quartile) had the highest frequency of postoperative hypotony (72%) compared with the eyes with the shortest AL (1st quartile, hypotony in 28% of eyes).

**Table 7:** Influence of axial length on the frequency of symptomatic hypotony. The range of axial length values was divided into four quartiles. The frequency of complications caused by ocular hypotony was obtained for each quartile group. Complications caused by hypotony were defined as choroidal swelling, shallow AC, and hypotonic maculopathy (choroidal and retinal folds, cystoid edema). *Only eyes, in which axial length measurement had been obtained, are listed*

	All eyes (*n* = 92)	1st quartile (*n* = 25)	2nd quartile (*n* = 23)	3rd quartile (*n* = 23)	4th quartile (*n* = 21)	*p* value
Range of axial length (mm)						
Frequency of hypotony	46 (50%)	7 (28%)	12 (52%)	12 (52%)	15 (72%)	0.032
Choroidal swelling	13 (14%)	2 (8%)	4 (17%)	4 (17%)	3 (14%)	
Shallow AC	4 (4%)	1 (4%)	1 (4%)	2 (9%)	0	
Hypotonic maculopathy	0	0	0	0	0	

(*mm*, millimetre; *AC*, anterior chamber)

### Analysis of risk factors for postoperative hypotony

In eyes with simultaneous cataract surgery, the risk for postoperative hypotony was about 0.5-fold (OR 0.483, 95% confidence interval 0.258–0.903).

Axial length over 24.3 mm (4th quartile) increases the risk for developing postoperative hypotony more than threefold (OR 3.226, 95% confidence interval 1.121–9.279). The following risk factors were not associated with a higher risk for hypotony: sex (OR 0.743, 95% confidence interval 0.404–1.367), arterial hypertension (OR 1.386, 95% confidence interval 0.752–2.556), primary versus secondary open-angle glaucoma (OR 1.242, 95% confidence interval 0.673–2.292), surgeries before XEN Gel Stent implantation (OR 1.163, 95% confidence interval 0.589–2.298), and injection of viscoelastic in the anterior chamber (OR 1.305, 95% confidence interval 0.639–2.663).

## Discussion

Glaucoma surgeries are judged based on their efficacy to lower the intraocular pressure as well as on their complication rate. In this retrospective, single-centre study, the success regarding IOP lowering, the frequency of postoperative hypotony, and complications were analysed in 170 eyes with XEN Gel Stent implantation. Postoperative hypotony below 6 mmHg was found in 57% of eyes and was associated with a longer axial length. Complications occurred only in hypotonic eyes.

Although the rate of numerical hypotony was relatively high, only relatively few eyes (9% of entire cohort) had symptomatic, persistent hypotony requiring treatment. After 12 months, only 2.2% of eyes had hypotonic IOP values.

Nearly 3 out of 4 eyes had a history of ocular surgery before the XEN Gel Stent implantation. This rate is relatively high, but can be explained by the fact that all surgeries were respected (even laser interventions). The rate of previous glaucoma surgeries was 12%. Furthermore, this study is a retrospective analysis of real-world data instead of a prospective study with strict exclusion of previously operated eyes.

In our study cohort, mean IOP was reduced from 24.6 ± 8.4 mmHg at baseline to 18.4 ± 10.2 mmHg at the 1-month follow-up visit as well as a reduction of IOP-lowering agents from 2.6 ± 1.3 to 0.5 ± 1.2. Thereby, the achieved IOP values were in the high-teens compared to the results ranging in the mid-teens reported by different XEN studies: the mean reduction of IOP was 25% after 4 weeks in our cohort, whereas e.g. Widder et al. [10] reported a 30% (by 7.5 to 16.8 mmHg postoperatively) reduction. Grover et al. [11] showed a mean reduction of IOP by 37% (by 9.2 to 15.9 mmHg postoperatively) after 12 months, and Theilig et al. [12] a reduction by 32% (by 7.9 to 16.6 mmHg postoperatively). However, the number of additional IOP-lowering eye drops differed between the studies: in our study, the patients received only 0.5 ± 1.2 eye drops on average after 4 weeks, whereas 0.9 ± 1.4 drops were used at the 1-month visit in the study by Theilig et al. [12].

After 1 year, the mean IOP reduction was 38% in our cohort, which is comparable to other studies: Grover et al. [11] showed a mean reduction of IOP by 37% after 12 months, and Theilig et al. [12] a reduction by 32%.

Ocular hypotony is usually defined by a numerical value, usually by values equal to or below 6 mmHg. However, hypotony-typical complications as macular folds or choroidal detachment can occur at higher IOP values as well, whereas some eyes can tolerate low IOP values without clinical signs. Abbas et al. [13] stated that we should attach more importance to the distinction between symptomatic and asymptomatic hypotony, rather than a numerical limitation.

In our study, we defined hypotony numerically as equal to 6 mmHg or below, and classified it in asymptomatic, symptomatic but transient (no treatment needed), and symptomatic (treatment needed). The symptoms of hypotony were choroidal swelling and shallow AC.

To our knowledge, there are not many studies in the literature about the frequency of hypotony and its reasons after XEN Gel Stent implantation.

Widder et al. [10] observed that complications such as AC bleeding and choroidal detachment resolved spontaneously after surgery. Furthermore, 3 of 233 eyes developed a shallow AC.

In our study cohort, choroidal swelling was found in 25 eyes and shallow AC in 4 eyes. We reported no cases of hypotonic maculopathy.

Karimi et al. found a hypotony rate of 34.7% after XEN Gel Stent implantation [7]. Seventy-two percent of the cases with hypotony showed a spontaneous resolution. Hypotony maculopathy occurred in 1.9% only, persisting choroidal effusion in 1.5%.

Theilig et al. [12] compared the temporary hypotony (defined as values ≤ 5 mmHg) between the TE group and XEN group. They reported a higher prevalence of hypotony in the XEN group (30%) than in the TE group (16%).

Significantly lower hypotony rate was already reported by Hengerer et al. [14]. In their retrospective analysis, they describe the safety and efficacy of the XEN Gel Stent. Hypotony was described in 9% of all eyes at the 4-week follow-up, 0.8% needing a surgical intervention.

Grover et al. [11] described in their multicentre clinical trial the postoperative complications after XEN Gel Stent implantation as moderate and transient. 16/65 patients (24.6%) had a transient hypotony < 6 mmHg without sequelae or the need for intervention. No cases of choroidal haemorrhage, choroidal effusion, or maculopathy were reported.

Recalling the anatomy of the eye, and the fact that anti-glaucomatous cataract surgery is often performed in angle-closure glaucoma, we compared the development of hypotony between the standalone XEN Gel Stent implantation (XEN group) and the combination of XEN Gel Stent and cataract surgery (combination group), in order to find a correlation between them. We hypothesized that probably a combination surgery would lead to a greater reduction of the IOP and therefore a greater hypotony-related complications rate.

Interestingly, the hypotony rate was significantly lower in the combination group (46.3%) than in the XEN group (64.1%, *p* = 0.027). No eyes with angle-closure glaucoma were included in this study.

The simultaneous cataract surgery can be interpreted as a protective factor against postoperative hypotony (OR 0.483). The reason for this unexpected finding is unclear.

There was no significant difference in the rates of shallow AC and choroidal swelling in the group of eyes, which were phakic at time of XEN Stent implantation compared with already pseudophakic eyes.

Karimi et al. [7] did not find any statistically significant difference in the development of complications between the two analysed groups (XEN standalone vs XEN with phacoemulsification).

To our knowledge, not many studies have been published about the risk factors of hypotony as well as the incidence of hypotony after MIGS implantation.

In our study, no significant difference was found in the analysed risk factors except for the axial length. The subgroup of eyes with longer axial length had a threefold risk for postoperative hypotony in comparison with smaller eyes. Surgeons should be aware of this risk factor for hypotony when counselling patients about the risks of the surgery.

The reason for the higher hypotony rate in longer eyes might be explained by the thinner scleral wall with potential leakage of aqueous humour adjacent to the XEN Gel Stent.

Limitations of our study comprise its retrospective setting and the follow-up period of 12 months only. The decision whether to observe the patient or to stabilize the anterior chamber with viscoelastic in case of hypotony was not clearly defined.

Another limitation of this study is the inclusion of both eyes of the same patient in the analysis. This is a possible bias, since risk factors might be over-represented if they occurred in a patient with bilateral XEN Gel Stent implantation. Nevertheless, we had decided to include both eyes in our evaluation since the cohort represents the real-world situation with bilateral surgery in 25% of patients.

The rate of postoperative hypotony in the second eye was not increased compared to the hypotony rate of the general cohort. Therefore, inclusion of both eyes in this analysis should not be considered as a bias regarding the frequency of postoperative hypotony.

From our study, we can conclude that the development of hypotony after XEN Gel Stent surgery was common in our sample, although in most cases, it occurs to be only numerical and asymptomatic or with spontaneous resolution. Furthermore, hypotony occurred most commonly within the first week of surgery. Since hypotony was observed mostly in myopic eyes, a XEN Gel Stent implantation might be reconsidered in eyes with a greater axial length, even though every single case should be properly discussed and estimated. Overall, the development of hypotony after XEN Gel Stent implantation does not have any long-lasting problematic consequence in the success or outcome parameters.

## Data Availability

All authors state that data and materials comply with field standards.

## Abbreviations

MIGS: Minimal invasive glaucoma surgery
IOP: Intraocular pressure
AL: Axial length
AC: Anterior chamber
BCVA: Best-corrected visual acuity
logMAR: Logarithm of the minimum angle of resolution
pOAG: Primary open angle glaucoma
PEX: Pseudoexfoliation
sOAG: Secondary open angle glaucoma
OR: Odds ratio
